# Protocol for genome-wide DNA replication timing analysis using click chemistry-based biotinylation

**DOI:** 10.1016/j.xpro.2025.104058

**Published:** 2025-09-03

**Authors:** Deniz Gökbuget, Kayla Lenshoek, Robert Blelloch

**Affiliations:** 1 The Eli and Edythe Broad Center of Regeneration Medicine and Stem Cell Research, Center for Reproductive Sciences, University of California, San Francisco, San Francisco, CA, USA; 2 Department of Urology, University of California, San Francisco, San Francisco, CA, USA; 3 Helen Diller Family Comprehensive Cancer Center, University of California, San Francisco, San Francisco, CA, USA

**Keywords:** Cell Biology, Genetics, Genomics, Molecular Biology

## Abstract

DNA replication timing (RT) is the cell-type-specific order by which different genomic regions are replicated during the S phase. Here, we present a biotinylation-based version of Repli-seq (BioRepli-seq) to determine genome-wide RT through next-generation sequencing. We detail steps for nucleotide analog pulse labeling, DNA content-based cell sorting, click chemistry-based biotinylation, DNA fragmentation, and on-bead sequencing library generation. This technique can be applied to any proliferating cell type to study RT and is compatible with automatization.

For complete details on the use and execution of this protocol, please refer to Gökbuget et al.^1^

## Highlights

• Procedures for EdU pulse labeling, fixation, and flow cytometric sorting
• Steps for DNA fragmentation and click chemistry-based biotinylation of nascent DNA
• Guidance on streptavidin pull-down and on-bead library preparation steps
• Instructions for data analysis, including RT calculation and segmentation

## Abstract (editor highlights)

Publisher’s note: Undertaking any experimental protocol requires adherence to local institutional guidelines for laboratory safety and ethics.

## Graphical abstract

**Figure:**
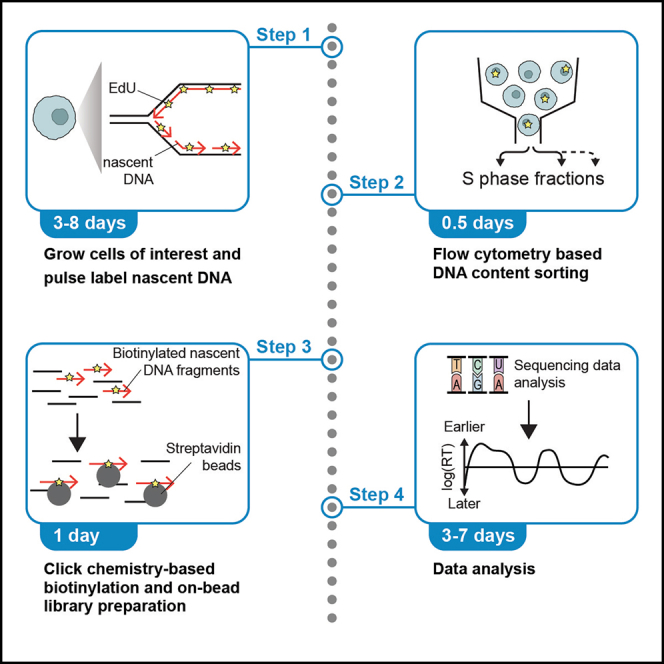

## Before you begin

**Figure 1:**
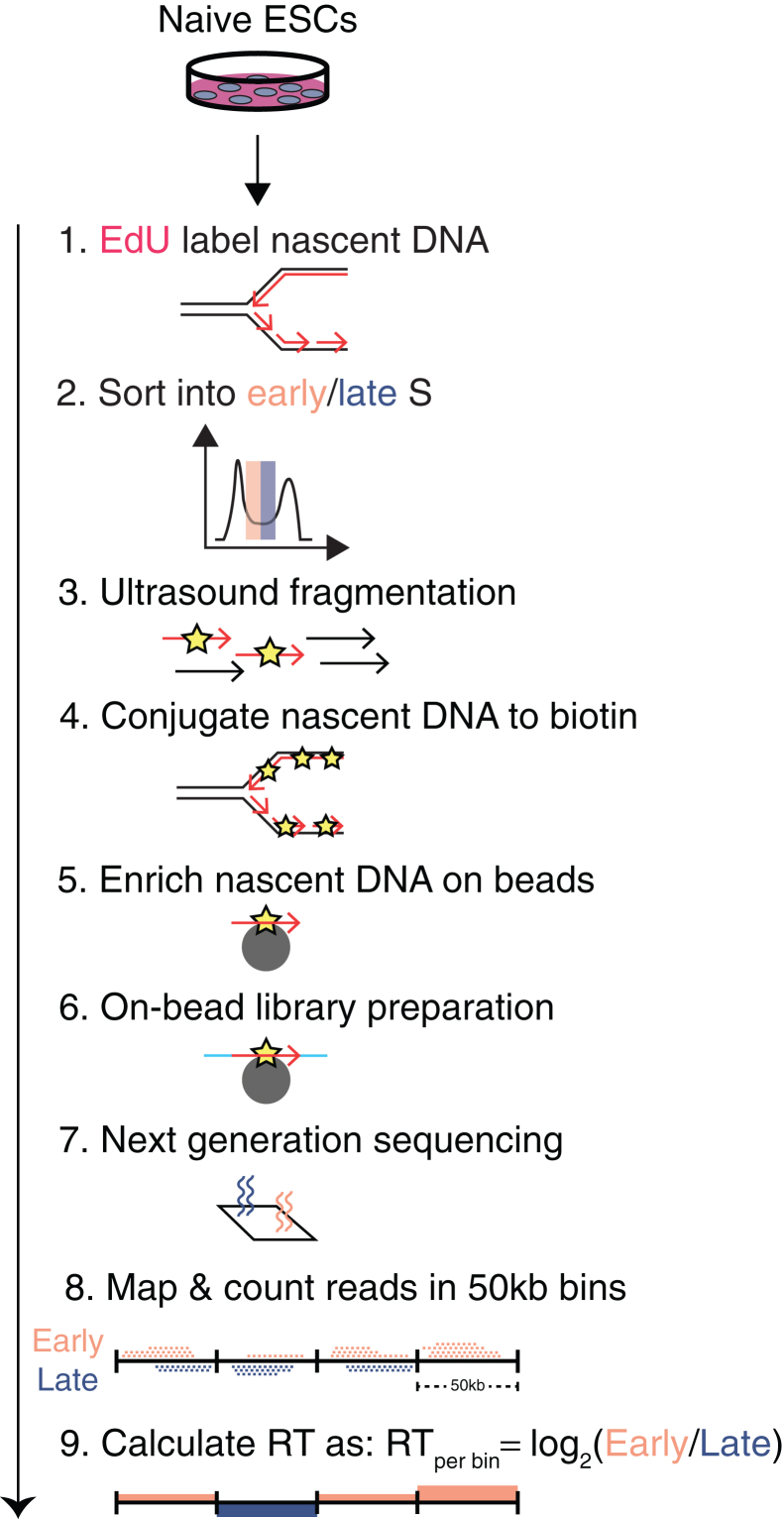
BioRepli-seq workflow

Profiling of genome-wide RT will advance our understanding of causal relationships driving chromatin functions such as gene expression. Previous techniques to determine genome-wide RT have relied on BrdU nucleotide incorporation, antibody-mediated enrichment of denatured BrdU-labeled DNA, DNA elution, and separate sequencing library preparation.^2^^,^^3^ However, the weaker antibody–antigen interaction in these methods dictates gentler (i.e., less stringent) washing conditions to avoid losing the BrdU-labeled DNA, often necessitating higher input cell numbers and making on-bead library preparation impractical. Consequently, these constraints can reduce the specificity of pulldown and limit the dynamic range of RT detection. To address these limitations, we present a streamlined technique leveraging the one-order-of-magnitude-stronger biotin–streptavidin interaction, enabling more stringent washes, reduced cell input, and efficient on-bead library preparation. We expect that this approach will also be compatible with liquid handling robotics for increased efficiency. A step-by-step overview is shown in Figure 1, and essential equipment/reagents can be found in the materials and equipment Setup section. In the workflow below, we used this technique to determine the genome-wide RT in mouse embryonic stem cells (ESCs) and correlated it with other chromatin features such as histone modifications and transcription to infer functional relationships.

## Key resources table

**Table:** 

REAGENT or RESOURCE	SOURCE	IDENTIFIER
**Chemicals, peptides, and recombinant proteins**		

Gelatine	MilliporeSigma	G1890
KO DMEM	Thermo Fisher Scientific	10829018
Fetal bovine serum	Corning	35-010-CV
NEAA	Thermo Fisher Scientific	11140035
L-glutamine	Thermo Fisher Scientific	59202C-100ML
Penicillin/streptomycin	MilliporeSigma	P4333-100ML
PD0325901	Axon Medchem	1408
CHIR99021	Axon Medchem	1386
ESGRO recombinant mouse LIF protein	MilliporeSigma	ESG1106
1× PBS	Thermo Fisher Scientific	10010023
EdU	Thermo Fisher Scientific	A10044
Trypsin-EDTA	Thermo Fisher Scientific	25200072
35 μm cell strainer	Corning	352235
Ethanol	Koptec	V1016
PI/RNase	Thermo Fisher Scientific	501121519
10× TBS	Thermo Fisher Scientific	J62938.K7
Biotin azide	Thermo Fisher Scientific	B10184
CuSO_4_	MilliporeSigma	C1297-100G
THPTA	MilliporeSigma	762342-100MG
Na-ascorbate	VWR	95035-692
Tris-HCl pH 7.5	Teknova	T5110
NaCl	Thermo Fisher Scientific	AM9760G
TE pH 8	Thermo Fisher Scientific	No.AM9849

**Critical commercial assays**		

NucleoSpin tissue kit	Macherey-Nagel	740952.50
Streptavidin beads	NEB	S1420S
NEBNext Ultra II	NEB	E7645S
Agencourt AMPure XP beads or SPRIselect beads	Beckman Coulter	A63880 or B23317
TapeStation D1000 tapes	Agilent	5067-5582
TapeStation High Sensitivity D1000 tapes	Agilent	5067-5584
TapeStation D1000 reagents	Agilent	5067-5583
High Sensitivity D1000 reagents	Agilent	5067-5585
microTUBE AFA sonication tubes	Covaris	520045
Qubit 1X dsDNA High Sensitivity (HS)	Thermo Fisher Scientific	Q33230

**Deposited data**		

Example BioRepli-seq data	Gökbuget et al.^1^	GEO: GSE216475

**Experimental models: Cell lines**		

V6.5 mouse embryonic stem cells	Novus Biologicals	NBP1-41162

**Software and algorithms**		

bowtie2	Langmead et al.^4^	https://github.com/BenLangmead/bowtie2
bedtools2	Quinlan et al.^5^	https://bedtools.readthedocs.io/en/latest/
DNAcopy	Olshen et al.^6^	https://bioconductor.org/packages/release/bioc/html/DNAcopy.html
Analysis code	Gökbuget^7^	https://github.com/dgoekbuget/2025-bioRepliSeq-StarProtocols

## Materials and equipment

**Table:** ESC media

Reagent	Final concentration	Amount
KO DMEM	N/A	410 ml
Fetal bovine serum	15%	75 ml
NEAA	1×	5 ml
L-Glutamine	1×	5 ml
Penicillin/Streptomycin	1×	5 ml
PD0325901∗	1 μM	N/A
CHIR99021∗	3 μM	N/A
ESGRO Recombinant Mouse LIF Protein∗	1000 U/ml	N/A
Total	N/A	500 ml

• Store media at 4°C for up to 3 months

**CRITICAL:** ∗To prevent degradation supplement two inhibitors (2i) PD0325901 and CHIR99021, as well as LIF daily to required media amount before adding to cells.

## Step-by-step method details

### Cell culture

**Timing: 3–7 days**

This step describes how to culture cells in preparation for nucleotide labeling.

1. Culture cells.
  In principle, this technique should work with any proliferating cell type. However, culture conditions, exponential growth window, and EdU labeling duration may need to be optimized.
  a. Coat one well of a 6-well plate per condition with gelatin.
    i. Add 1 ml of 0.2% gelatin solution per well.
    ii. Incubate for 10 min at room temperature.
  b. Aspirate gelatin and seed 2×10^4^ cells per condition per well in 2 ml media and distribute cells evenly by gentle agitation.
    Aim for at least 1×10^6^ cells per sample at day of intended labeling (step 2), although the protocol could likely be optimized to work with less cells.
    ***Optional:*** Prepare an extra sample to optimize gating for flow cytometry sorting.
  c. Incubate cells at 37°C/5% CO_2_ until they achieve desired density with daily media changes.
    Approximately 48 h will typically yield the desired minimum of 5x10^5^ cells per sample for proceeding to next step.

### EdU labeling and ethanol fixation

**Timing: 1.5 days**

This step describes the nucleotide labeling, cell harvesting, and fixation procedures.

2. Label nascent DNA with EdU.
  **CRITICAL:** Cells need to be in exponential growth phase for this step to ensure active proliferation. If unsure, perform a growth curve first to determine the ideal time point for labeling.
  a. Equilibrate fresh media.
    Prepare an empty culture dish with 1 ml media per condition and incubate under same conditions at 37°C/5% CO_2_ for 1 h.
  b. Add EdU to equilibrated media and mix well by pipetting.
    The final concentration of EdU should be 100 μM. Keep the media in the incubator until you are ready for the next step.
  c. Aspirate media from cells and immediately add EdU-containing media.
    The incubation time may need to be optimized for other cell types to ensure sufficient labeling of nascent DNA and to cover the full length of S phase.
  d. Incubate cells at 37°C/5% CO_2_ for exactly 2 h.
    **CRITICAL:** To ensure precise labeling conditions, labeling of more than 8–12 samples at a time is discouraged. Additionally, it is recommended to start and stop labeling at fixed intervals. For example, begin labeling sample 1, then 30 s later begin labeling sample 2, and stop them in the same 30 s increments. This labeling time has been optimized for ESCs and may need to be optimized for other cell types with different S phase durations.
  e. Aspirate media and immediately add 2 ml ice-cold PBS to each well.
    **CRITICAL:** To prevent any ongoing DNA replication, keep cells ice cold at all times, except during the trypsinization step (f).
  f. Aspirate PBS, add enough trypsin solution to cover cells.
  g. Incubate cells at 37°C for 2 min.
    Trypsinization duration needs to be optimized for different cell types.
  h. Immediately, quench trypsin by adding 2 ml ice-cold media and resuspend cells gently into single cell suspension.
  i. Filter cells through strainer into FACS tube and spin at 300×g for 3 min at RT.
  j. Discard the supernatant and wash the cells with ice-cold PBS.
    Ensure not to disturb pellet when discarding supernatant. Spin at 300×g for 3 min for washing.
    ***Optional:*** Take an aliquot and ensure viability is > 90% (e.g., trypan blue staining).
  k. Discard the supernatant and resuspend the cells in 300 μl ice-cold PBS.
3. Ethanol fixation (day 3).
  a. Turn on vortex mixer at intermediate settings and place tube with cells
  b. While mixing, add 700 μl of pure ethanol (cooled at −20°C) dropwise to cells.
    **Pause point:** Fix cells at −20°C overnight.

If necessary, this fixation can be extended to 3 days.

### DNA content-based cell sorting and genomic DNA extraction

**Timing: 6 h**

This step describes how to stain DNA and subsequently perform flow cytometry based cell sorting based on DNA content.

4. Stain cellular DNA and sort cells into S phase fractions.
  a. Wash fixed cells once with 1% BSA/PBS at RT.
    Perform spins at 500×g for 3 min for washing.
  b. Resuspend cell pellet in 500 μl of PI/RNase staining solution.
  c. Incubate at RT in the dark for 20 min.
  d. Place tubes on ice and sort 60,000 cells each into early and late S phase fractions.
    Use linear scale for PI channel on flow cytometer and draw two equally-sized sorting gates ranging from the G1 phase peak (2n) to the middle of S phase and from the middle of S phase to the G2/M (4n) phase peak. It is possible to sort more S phase fractions to increase genomic resolution of RT.
  e. Keep sorted cells on ice until further processing.
  f. Extract genomic DNA per instructions of the NucleoSpin Tissue kit.
  g. Elute in 100 μl of H_2_O.
    **Pause point:** Store samples at −20°C or proceed to next step.

### DNA fragmentation

**Timing: 2 h**

This step describes how to perform sonication-based DNA fragmentation.

5. Fragment DNA into approximately 200 bp sized fragments.
  a. Cool down and degas sonicator.
  b. Transfer 50 μl of genomic DNA to a sonication tube and keep on ice.
  c. Sonicate on Covaris sonicator using settings below and keep samples on ice.
    Ideal settings have to be experimentally determined if using a different sonicator.
    **Table:** Sonication settings

Peak Incidence Power	175 W
Duty Factor	10
Cycles Per Burst	200
Temperature	4°C
Time	90 s
  d. Quickly spin down sonication tubes and transfer fragmented DNA to PCR tube.
  e. Optional: Use 1 μl of DNA to validate fragmentation using Tapestation with D1000 High Sensitivity reagents or equivalent analyzer.
    **Pause point:** Freeze samples at −20°C or proceed to next step.

### Biotinylation of EdU-labeled DNA

**Timing: 2 h**

This step describes how to covalently couple biotin to EdU-incorporated DNA fragments using click chemistry.

6. Biotinylation of EdU-labeled DNA through click chemistry.
  a. Prepare 1:1 Cu/THPTA mixture as shown below.
    **Table:** Cu/THPTA mixture

Reagent	Per reaction (add 10% excess)
10 mM CuSO4	0.65 μl
50 mM THPTA	0.65 μl
  b. Freshly prepare 6.5 μl 100 mM sodium ascorbate per sample (add 10% excess).
    100 mM sodium ascorbate equals a concentration of approximately 20 mg/ml.
  c. Pipette click reaction in the order shown below.
    **Table:** Click reaction

Reagent	Per reaction
Fragmented DNA	50 μl
10× TBS	6.5 μl
1 mM Biotin azide	0.65 μl
Cu/THPTA mixture	1.3 μl
100 mM Sodium ascorbate	6.5 μl
  d. Incubate for 1 h at RT protected from light.
  e. Perform DNA fragment size selection.
  f. Prepare 500 μl of fresh 80% EtOH per sample.
  g. Add 0.5 volumes of SPRI beads and mix well by pipetting up and down 10×.
  h. Incubate 5 min at RT.
  i. Incubate on magnet for 2–3 min or until solution appears clear.
  j. Transfer supernatant to new PCR tubes.
  k. Add 0.4 volumes of SPRI beads.
  l. Incubate 5 min at RT.
  m. Incubate on magnet for 2–3 min or until solution appears clear.
  n. Wash 2× with 200 μl 80% EtOH on magnet.
    Incubate approximately 30 s each wash step.
  o. Dry beads 3–5 min.
    Do not over dry beads as this can negatively affect yield.
  p. Elute beads by adding 51 μl 0.1× TE and incubating for 2 min at RT.
  q. Incubate on magnet for 2–3 min or until solution appears clear.
  r. Transfer 50 μl of supernatant to new PCR tubes.
  s. Determine DNA concentration.
  t. Use 1 μl of sample to determine DNA concentration per instructions of the Qubit DNA kit.
    **Pause point:** Freeze samples at −20°C or proceed to next step.

### Streptavidin pull-down and on-bead sequencing library preparation

**Timing: 6 h**

This step describes the on-bead sequencing library generation.

7. Enrich biotinylated DNA by streptavidin pulldown.
  a. Heat 2× B&W buffer in water bath to 55°C.
  b. Mix 49 μl of streptavidin beads per sample and wash twice in 2× B&W buffer.
    Wash by placing beads on magnet until clear and gently resuspend in 2× B&W buffer by pipetting. Resuspend washed beads in 49 μl 2× B&W buffer per sample (add 10% excess).
  c. Use approximately 25–50 ng of size-selected DNA as input and adjust volume to 49 μl using 0.1× TE.
  d. Add 49 μl of washed SA beads to DNA and mix by pipetting up and down 10×.
  e. Incubate 15 min at RT.
  f. Wash for 2 min with 200 μl heated 2× B&W.
  g. Incubate on magnet for 2–3 min or until solution appears clear.
  h. Repeat wash and magnet steps once (f and g).
  i. Wash with 100 μl 0.1× TE.
  j. Incubate on magnet for 2–3 min or until solution appears clear.
  k. Discard supernatant and resuspend beads in 25 μl 0.1× TE.
  l. Immediately proceed to next step.
8. Perform End prep.
  a. Pipette End prep reaction by mixing the following reagents.
    **Table:** End prep reaction

Reagent	Per reaction
Bead-enriched DNA	25 μl
End Prep Reaction Buffer	3.5 μl
End Prep Enzyme Mix	1.5 μl
  b. Mix well by setting a pipette to 15 μl and pipetting up and down 10×.
  c. Incubate on a PCR machine using the following settings.

**Table:** End prep PCR machine settings

Steps	Temperature	Time
1	20°C	30 min
2	65°C	30 min
3	4°C	Hold

9. Immediately perform ligation.
  a. Pipette ligation reaction by mixing the following reagents.
    **Table:** Ligation reaction

Reagent	Per reaction
End prepped DNA	30 μl
Ligation Master Mix^a^	15 μl
Ligation Enhancer	0.5 μl
Adaptor for Illumina (1:10)^b^	1.25 μl

a Mix well by pipetting before adding.

b Dilute in Tris/NaCl solution (1 mM Tris pH8, 10 mM NaCl).
  b. Mix well by setting a pipette to 20 μl and pipetting up and down 10×.
  c. Incubate on a PCR machine using the following settings.
    **Table:** Ligation PCR machine settings

Steps	Temperature	Time
1	20°C	15 min
2	4°C	Hold
  d. Add 1.5 μl USER Enzyme and mix thoroughly by pipetting.
  e. Incubate in PCR machine at 37°C for 15 min (lid set to > 45°C).
10. Wash beads and elute adapter-ligated DNA from beads.
  a. Incubate beads on magnet for 2–3 min or until solution appears clear.
  b. Discard supernatant.
  c. Wash for 2 min with 200 μl heated 2× B&W.
  d. Incubate on magnet for 2–3 min or until solution appears clear.
  e. Repeat wash and magnet steps once (c and d).
  f. Wash with 100 μl 0.1× TE.
  g. Incubate on magnet for 2–3 min or until solution appears clear.
  h. Discard supernatant and resuspend beads in 8 μl 0.1× TE.
  i. Immediately proceed to next step.
  j. Elute by incubating beads in PCR machine at 98°C for 10 min (lid set to 105°C).
  k. Incubate on magnet for 2–3 min or until solution appears clear.
  l. Transfer 7.5 μl of supernatant to new PCR tube.
11. Perform indexing PCR amplification.
  a. Pipette PCR reaction by mixing the following reagents.
    **CRITICAL:** Make sure to use one unique i7 index primer per sample and avoid any cross-contamination. The universal i7 index primer is provided to all samples.
    **Table:** Indexing PCR reaction

Reagent	Per reaction
Eluted adapter-ligated DNA	7.5 μl
NEBNext Ultra II Q5 Master Mix	12.5 μl
Universal i7 primer	2.5 μl
Unique i7 primer (per sample)	2.5 μl
  b. Mix well by setting a pipette to 10 μl and pipetting up and down 10×.
  c. Incubate on a PCR machine using the following settings (with lid set to 105°C).

**Table:** Indexing PCR settings

Steps	Temperature	Time	Cycles
Initial Denaturation	98°C	30 s	1
Denaturation	98°C	10 s	14 cycles^a^
Annealing & Extension	65°C	20 s	
Final extension	65°C	5 min	1
Hold	4°C	∞	

a Cycle number depends on input. If using more or less input, adjust the number of cycles proportionally. Aim for > 5 nM of amplified DNA per sample.

12. Immediately proceed to SPRI bead enrichment.
  a. Prepare 500 μl of fresh 80% EtOH per sample.
  b. Add 0.7 volumes of SPRI beads and mix well by pipetting up and down 10×.
  c. Incubate 5 min at RT.
  d. Incubate on magnet for 2–3 min or until solution appears clear.
  e. Discard supernatant and incubate on magnet for 2–3 min or until solution appears clear.
  f. Wash 2x with 200 μl 80% EtOH on magnet.
    Incubate approximately 30 s each wash step.
  g. Dry beads 3–5 min.
    Do not over dry beads as this can negatively affect yield.
  h. Elute beads by adding 21 μl 0.1× TE and incubating for 2 min at RT.
  i. Incubate on magnet for 2–3 min or until solution appears clear.
  j. Transfer 20 μl of supernatant to new PCR tubes.
    **Pause point:** Freeze at −20°C or proceed to next step.
13. Validate fragment size and determine concentration using Tapestation with D1000 reagents per instruction by the manufacturer or equivalent.

A concentration of > 5 nM for each sample is required for Illumina sequencing. Make sure the fragment size distribution has an average of 400–600 bp. Determine concentration using region settings between 100-900 bp.

### Sequencing

**Timing: 1–2 weeks**

This step describes how to combine individual libraries in equimolar amounts for sequencing and specifies recommended sequencing parameters.

14. Perform stoichiometric sample pooling and send samples out for sequencing.
  a. Pool samples in equimolar stoichiometries at a final concentration of > 5 nM in at least 20 μl.
  b. Send samples to sequencing facilities and sequence >10M paired-end reads per sample on an Illumina flow cell.

Any read length larger than 50 bp should work with this technique with no changes in the data analysis pipeline. Single-end read sequencing is expected to be compatible with this technique as well but with minor adjustments to the bowtie2 mapping step to execute singe read mapping.

### Data analysis

**Timing: 3–7 days**

Data analysis steps include quality control, mapping, calculation of RT, and RT segmentation, and were adapted from previous workflows.^3^

***Note:*** With greater sample sizes it is recommended to parallelize the code below for increased efficiency using common job schedulers like Sun Grid Engine or Portable Batch System.

15. Perform quality control of sequencing data.
  a. Use FastQC to assess quality of sequencing reads by running the following command in the folder containing sequencing .fastq files.

FastQC ∗.fastq

16. Map reads to the mouse genome and remove low-quality reads.
  a. Perform mapping with bowtie2^4^ to the mm10 mouse genome by running the following command for each pair of paired-end .fastq.gz files. Create bowtie2 index as per manual for mm10 genome.

bowtie2 -x '/path/to/bowtie2index' --no-mixed --no-discordant --reorder -X 1000 -1 mate1.fastq.gz -2 mate2.fastq.gz -S mapped.sam 2>> mapped.mapping.statistics.txt

samtools view -bSq 20 mapped.sam > mapped.bam

samtools sort -o mapped.sorted.bam mapped.bam

samtools rmdup -S mapped.sorted.bam mapped.deduplicated.bam

17. Calculate read density across non-overlapping genomic bins.
  a. Create .bed file for mm10 genome with non-overlapping 50 kb bins.
    Optimal bin size depends on the depth of sequencing and should be optimized.
    bedtools makewindows -w 50000 -s 50000 -g mm10.genome.chrom.sizes > mm10.genome.50kb.bins.bed
  b. Calculate read density per 50 kb bin in counts per million (Cpm) for each early and late fraction mapped deduplicated .bam files (step 2).

for file in "∗.early.bam" "∗.late.bam"; do

  bamToBed -i "$file" | cut -f 1,2,3,4,5,6 | sort -T . -k1,1 -k2,2n -S 5G > "${file%.bam}".bed

  x=`wc -l "${file%.bam}".bed | cut -d' ' -f 1`

  bedtools intersect -sorted -c -b "${file%.bam}".bed -a mm10.genome.50kb.bins.bed | awk -vx=$x '{print $1,$2,$3,$4∗1e+06/x}' OFS='\t' > "${file%.bam}".bedGraph

  done

18. Calculate RT for each sample as log2(early/late) and merge into tab-separated table.

for file in ∗.early.bedGraph

do paste $file "${file%.early.bedGraph}".late.bg | awk '{if($8 != 0 && $4 != 0){print $1,$2,$3,log($4/$8)/log(2)}}' OFS='\t' > ${file%.early.bedGraph}.RT.bedGraph

done

echo -e "chr\tstart\tstop\t"`ls ∗RT.bedGraph` | sed 's/\ /\t/g' > merge_RT.txt

bedtools unionbedg -filler "NA" -i ∗RT.bedGraph >> merge_RT.txt

19. Perform quantile normalization and loess smoothing in R.
  a. Load required packages.
    library(readr)
    library(preprocessCore)
    library(DNAcopy)
  b. Define file paths and perform quantile normalization.
    #Define file paths and other parameters
    setwd("/path/to/workdir") #Working directory. Needs to contain "merge_RT.txt" (step 4).
    #Import and prepare RT data
    merge <- read_delim("merge_RT.txt", delim = "\t",
    escape_double = FALSE,
    trim_ws = TRUE)
    merge_values <- as.matrix(merge[,4:ncol(merge)])
    ad <- stack(merge[,4:ncol(merge)])$values
    norm_data <- normalize.quantiles.use.target(merge_values,ad)
    merge_norm <- data.frame(merge[,1:3],norm_data)
    colnames(merge_norm) <- colnames(merge)
  c. Perform loess smoothening.
    dat <- merge_norm
    dat <- dat[complete.cases(dat),]
    ind <- c(grep("chrM",dat$chr),grep("chrY",dat$chr)) #Remove Y and mitochondrial chromosomes from analysis
    dat <- dat[-ind,]
    colnames(dat)[4:ncol(dat)] <- gsub(".bg","",colnames(dat)[4:ncol(dat)])
    dat$start <- as.integer(dat$start)
    dat$stop <- as.integer(dat$stop)
    #Perform Loess smoothening and export bedgraph files for plotting
    #Generate quantile normalized bedgraphs per sample
    for(i in 4:ncol(merge_norm)){write.table(merge_norm[complete.cases(merge_norm[,i]), c(1,2,3,i)],
     gsub(".bg" , ".qnorm.bg", colnames(merge_norm)[i]),
     sep= "\t" ,row.names=FALSE, quote=FALSE, col.names = FALSE)
    }
    # Perform loess smoothing
    ind <- c(grep("chrM",merge_norm$chr),grep("chrY",merge_norm$chr))
    chrs=unique(merge_norm$chr[-ind])
    AllLoess=list()
    for(i in 1:(ncol(merge_norm)-3)){
           AllLoess[[i]]=data.frame();
           cat("Current dataset:", colnames(merge_norm)[i+3], "\n");
           for(Chr in chrs){
       RTb=subset(merge_norm, merge_norm$chr==Chr);
       lspan=300000/(max(RTb$start)-min(RTb$start));
       cat("Current chrom:" , Chr, "\n");
       RTla=loess(RTb[,i+3] ∼ RTb$start, span=lspan);
       RTl=data.frame(c(rep(Chr,times=RTla$n)), RTla$x, merge_norm[which( merge_norm$chr==Chr & merge_norm$start %in% RTla$x),3],RTla$fitted);
       colnames(RTl)=c("chr" , "start" , "end" ,colnames(RTb)[i+3]);
       if(length(AllLoess[[i]])!=0){
         AllLoess[[i]]=rbind(AllLoess[[i]],RTl)
       } else if(length(AllLoess[[i]])==0){
        AllLoess[[i]] = RTl
       }
    }
    #Write quantile normalized bedgraphs per sample
    for(i in 1:length(AllLoess)){write.table(AllLoess[[i]][complete.cases(AllLoess[[i]]),],
      gsub(".bedGraph" , ".Loess.bedGraph" , colnames(AllLoess[[i]]))[4], sep= "\t",
      row.names=FALSE, quote=FALSE, col.names = FALSE)
    }
  d. Merge loess-smoothened normalized RT into a tab-separated table by running the following code in your directory containing exported .Loess.bedGraph files (step c).
    echo -e "chr\tstart\tstop\t"`ls ∗RT.bedGraph` | sed 's/\ /\t/g' > merge_Loess_norm_RT.txt
    bedtools unionbedg -filler "NA" -i ∗Loess.bedGraph >> merge_Loess_norm_RT.txt
  e. Optional: Convert obtained .bedgraph files into more lightweight .bigwig files for faster and more versatile plotting in genome browsers such as IGV^8^ or UCSC Genome Browser.^9^
    bedGraphToBigWig "input.bedGraph" mm10.chrom.sizes "output.bigwig"
  f. Optional: Assess reproducibility of replicates through correlation analysis.
20. Segmentation of RT bins.
  a. Load table with loess-smoothened normalized RT values for each sample into R, filter out missing data, and adjust formatting.
    dat_loess <- data.frame(read_delim("merge_Loess_norm_RT.txt", delim = "\t",escape_double = FALSE, trim_ws = TRUE))
    dat_loess <- dat_loess[complete.cases(dat_loess),]
    colnames(dat_loess)[4:ncol(dat_loess)] <- gsub(".bg","",colnames(dat_loess)[4:ncol(dat_loess)])
    dat_loess$start <- as.integer(dat_loess$start)
    dat_loess$stop <- as.integer(dat_loess$stop)
  b. Optional: Average replicates.
  c. Use circular binary segmentation algorithm^6^ to unbiasedly combine RT bins of a given sample into segments.
    #Circular binary segmentation
    library(DNAcopy)
    library(rafalib)
    set.seed(178)
    #Empiric determination of optimized segmentation parameters
    input <- dat_loess[1:1000,] #Choose a custom window of consecutive RT bins here
    mypar(4,5)
    dat.cna = CNA(input$mysample.averageRT, input$chr, input$start, data.type = "logratio",
      sampleid = "My Sample")
    for (i in seq(1,20,by=2)) {
     for(j in c(1e-15,1e-200)){
     seg.cna = segment(dat.cna, nperm = 1000, alpha = j, undo.splits = "sdundo",
       undo.SD = i, verbose = 0)
     plot(subset(seg.cna,chromlist = "chr1"), pch = 19,
      pt.cols = c("gray","gray"), xmaploc = T, ylim = c(-5,5),
      )
     legend("topright",legend=paste0("alpha=",j," undo.SD=",i),cex=0.8,box.lty=0,bg="transparent")
     }
    }
  d. Evaluate plots to choose optimal segmentation parameters. Note that the range of undo.splits and undo.SD parameters may have to be adjusted based on the data.
  e. Perform segmentation of the whole genome with selected parameters.
    #Segmentation of averaged RT of sample of choice
    input <- dat_loess
    undo.SD=5 #choose optimized parameter here
    alpha=1e-15 #choose optimized parameter here
    dat.cna = CNA(input$mysample.averageRT, input$chr, input$start, data.type = "logratio", sampleid = "My Sample")
    seg.cna = segment(dat.cna, nperm = 10000, alpha = alpha, undo.splits = "sdundo", undo.SD = undo.SD, verbose = 2)
  f. Resulting object contains identified segments together with their location and average RT signal, which can be used for downstream analyses.

## Expected outcomes

**Figure 2:**
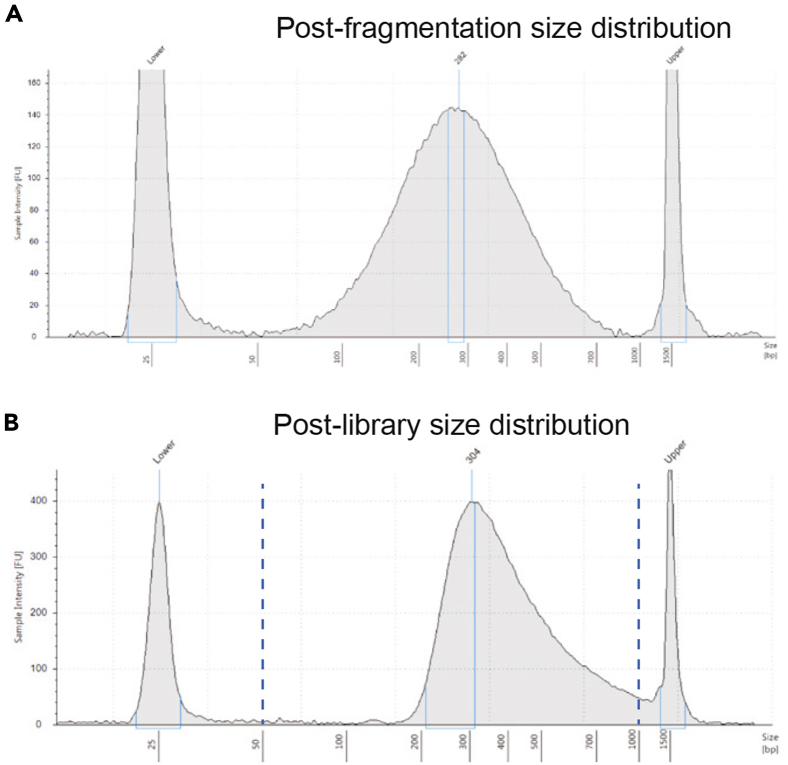
Example DNA size distribution results (A and B) Tapestation DNA size distribution for samples after DNA fragmentation step (A) and after library preparation (B).

**Figure 3:**
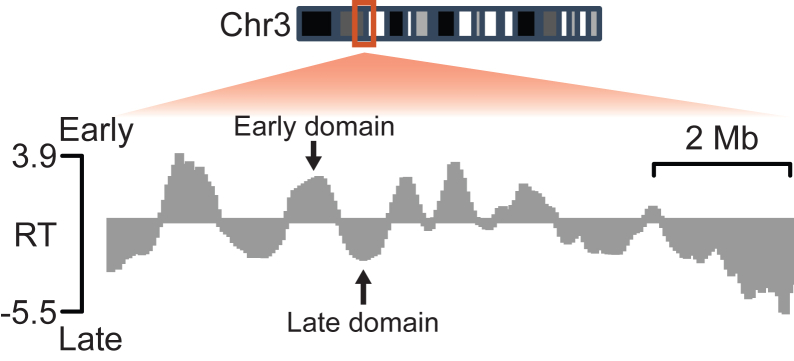
Example genome track result

After EdU-pulse labeling and DNA content staining, 60,000 cells will be sorted into early and late S phase fractions. Extracted genomic DNA will be fragmented to an average size of approximately 200 bp (Figure 2A). Following biotinylation, the expected input to biotin enrichment and library preparation is 25 to 50 ng. Library construction should yield a concentration of at least 5 nM with a size distribution centered at 400 to 600 bp (Figure 2B). Sequenced reads should overwhelmingly (> 95%) map to the mouse genome and show high reproducibility across replicates (Spearman’s rho > 0.98). Computational determination of RT should provide genome-wide RT with clearly defined early and late-replicating domains (Figure 3).

## Limitations

This method is intrinsically a bulk assay and thus cannot resolve replication timing at the single-cell level, so populations of interest should be relatively homogeneous. If mixed or heterogeneous lineages are present, each subpopulation should be characterized and processed separately, as pooling risks losing resolution of their distinctive replication timing profiles. Furthermore, it requires a sufficient number of cells to ensure robust DNA recovery; this can pose a challenge for rare or slowly proliferating populations. Successful EdU labeling depends on cells actively traversing S phase during the pulse, which may necessitate pilot studies to optimize pulse length or EdU concentration, particularly for cell types with prolonged or irregular S phases. It should also be noted that extended labeling times should be avoided as EdU-induced cytotoxicity has been reported 24 hours after labeling.^10^

## Troubleshooting

### Problem 1

DNA content staining failed (see DNA content-based cell sorting).

### Potential solution

Too many cells (>2×10^6^) for 500 μl PI staining solution may result in non-saturating staining conditions. Decrease input cell number to 10^6^ or increase PI staining solution volume.

### Problem 2

G2/M phase peak not detected (see DNA content-based cell sorting).

### Potential solution

Ensure the EdU concentration used is accurate. High concentrations can inhibit DNA replication and arrest cell cycle progression. Confirm cell viability is > 90% after harvesting. Dead or dying cells can skew results.

### Problem 3

DNA fragmentation failed or absent DNA (see DNA fragmentation).

### Potential solution

Ensure genomic DNA extraction was successful by measuring absorbance at 260 nm. Ensure that no air bubbles are introduced when dispensing genomic DNA to sonication tubes. Also, ensure that sonicator is degassed and water level is appropriate for the used tubes (see manufacturer’s instructions).

### Problem 4

No library detected (see library preparation).

### Potential solution

This could be due to issues in the biotinylation step or insufficient PCR amplification. Biotinylation of DNA can be validated through dot blots compared to a control omitting the biotin-azide reagent. To increase library yield, if possible, use more cellular input or alternatively increase number of PCR cycles. Also, ensure that beads are not lost or dried out during the washing steps of the library preparation.

### Problem 5

No RT domains detected (see data analysis).

### Potential solution

This could potentially be due to improper gating of S phase resulting in inefficient sorting. Ensure that early and late S phase fraction gates are non-overlapping and that sorting efficiency is high. Validate successful sorting by counting retrieved cells.

## Resource availability

### Lead contact

Requests for further information should be directed to and will be fulfilled by the lead contact, Deniz Goekbuget (deniz.goekbuget@ucsf.edu).

### Technical contact

Further information on technical details or requests regarding reagents should be directed to and will be fulfilled by the technical contacts, Deniz Goekbuget (deniz.goekbuget@ucsf.edu) and Robert Blelloch (robert.blelloch@ucsf.edu).

### Materials availability

All reagents are commercially available.

### Data and code availability

• Raw and processed BioRepli-seq data have been deposited at NCBI GEO and are publicly available as of the date of publication. Accession numbers are listed in the key resources table.
• Original code used in this study is publicly available through GitHub as of the date of publication. The link is available in the key resources table.
• Any additional information required to reanalyze the data reported in this paper is available from the lead contact upon request.

## Acknowledgments

Technical support by the UCSF Parnassus Flow Cytometry Core and the UCSF Center for Advanced Technology is greatly appreciated. This publication includes data generated at the UC San Diego IGM Genomics Center utilizing an Illumina NovaSeq 6000 that was purchased with funding from a National Institutes of Health SIG grant (#S10 OD026929). This research was supported by NIGMS funding to R.B. (R01GM122439 and R01GM125089).

## Author contributions

Conceptualization, D.G. and R.B.; methodology, D.G.; validation, D.G.; formal analysis, D.G.; investigation, D.G. and K.L.; resources, R.B.; data curation, D.G.; writing – original draft, D.G.; writing – review and editing, D.G. and R.B.; visualization, D.G.; supervision, D.G. and R.B.; project administration, R.B.; funding acquisition, R.B.

## Declaration of interests

The authors declare no competing interests.

## References

[1] Gökbuget D., Goehring L., Boileau R.M., Lenshoek K., Huang T.T., Blelloch R. (2025). KMT2C/KMT2D-dependent H3K4me1 mediates changes in DNA replication timing and origin activity during a cell fate transition. Cell Rep..

[2] Ryba T., Battaglia D., Pope B.D., Hiratani I., Gilbert D.M. (2011). Genome-scale analysis of replication timing: from bench to bioinformatics. Nat. Protoc..

[3] Marchal C., Sasaki T., Vera D., Wilson K., Sima J., Rivera-Mulia J.C., Trevilla-García C., Nogues C., Nafie E., Gilbert D.M. (2018). Genome-wide analysis of replication timing by next-generation sequencing with E/L Repli-seq. Nat. Protoc..

[4] Langmead B., Salzberg S.L. (2012). Fast gapped-read alignment with Bowtie 2. Nat. Methods.

[5] Quinlan A.R., Hall I.M. (2010). BEDTools: a flexible suite of utilities for comparing genomic features. Bioinformatics.

[6] Olshen A.B., Venkatraman E.S., Lucito R., Wigler M. (2004). Circular binary segmentation for the analysis of array-based DNA copy number data. Biostatistics.

[7] Gökbuget D. (2025).

[8] Robinson J.T., Thorvaldsdóttir H., Winckler W., Guttman M., Lander E.S., Getz G., Mesirov J.P. (2011). Integrative genomics viewer. Nat. Biotechnol..

[9] Perez G., Barber G.P., Benet-Pages A., Casper J., Clawson H., Diekhans M., Fischer C., Gonzalez J.N., Hinrichs A.S., Lee C.M. (2025). The UCSC Genome Browser database: 2025 update. Nucleic Acids Res..

[10] Haskins J.S., Su C., Maeda J., Walsh K.D., Haskins A.H., Allum A.J., Froning C.E., Kato T.A. (2020). Evaluating the Genotoxic and Cytotoxic Effects of Thymidine Analogs, 5-Ethynyl-2’-Deoxyuridine and 5-Bromo-2’-Deoxyurdine to Mammalian Cells. Int. J. Mol. Sci..

